# Molecular Characterization of *Borrelia burgdorferi* from Case of Autochthonous Lyme Arthritis

**DOI:** 10.3201/eid2012.140655

**Published:** 2014-12

**Authors:** Sharon I. Brummitt, Alan G. Barbour, Fong Hue, Anne M. Kjemtrup

**Affiliations:** County of Santa Cruz Communicable Disease, Santa Cruz, California, USA (S.I. Brummitt);; California Department of Public Health, Sacramento, California, USA (S.I. Brummitt., A.M. Kjemtrup);; University of California Irvine, Irvine, California, USA (A.G. Barbour, F. Hue)

**Keywords:** Lyme arthritis, Lyme disease California, ospC molecular characterization, *Borrelia burgdorferi*, bacteria, vectorborne, tickborne, tick, *Ixodes pacificus*, sensu lato, *recA* gene

**To the Editor**: The first Lyme borreliosis (LB) case reported to be acquired in California occurred in 1978 (*1*). During the past 10 years, 744 confirmed LB cases were reported in California; 419 (56.2%) were likely acquired in-state. The highest incidence of this disease occurs in northern coastal California, in locations such as Santa Cruz County (*2*), where habitat supports yearlong activity of the tick vector *Ixodes pacificus* (*3*,*4*).

Existing data describe the genetic diversity of the LB agent *Borrelia burgdorferi* among ticks in Californa (*5*,*6*), but few instances of direct detection and genetic characterization of *B. burgdorferi* sensu stricto in samples from humans are documented in California. *B. burgdorferi* has been isolated from skin biopsy samples of 3 patients in California in whom LB was diagnosed (*1*). Seinost et al. genotyped strains isolated in the United States, including 7 isolates identified in California from skin, blood, or cerebrospinal fluid, but no documented exposure information was available (*7*). Girard et al. genotyped *B. burgdorferi* in 10- to 12-year-old stored serum samples collected from 22 northern California residents, some of whom were asymptomatic at time of collection. Of 22 PCR-positive specimens, 21 had the single laboratory type strain B31 genotype (*3*).

A 12-year-old resident of Santa Cruz County, California, came to the emergency department of Dominican Hospital in September 2012 with a swollen, painful right knee and mildly painful right hip. The patient’s family reported that LB had been diagnosed by a local physician. Illness onset was in May 2010; symptoms consisted of recurrent knee swelling and pain lasting several days every 4–5 months and positive serologic test results for *B. burdorferi* (not available). The patient had not traveled outside of California during the preceding 6 years. In May 2011, an IgG Western blot of the patient’s serum that was processed at a commercial laboratory showed immunoreactive bands of 18, 23, 28, 30, 39, 41, 45, 58, 66, and 93 kDa. In both 2010 and 2011, the patient’s family had chosen to give the patient unspecified herbal treatments instead of antibacterial drugs.

On physical examination in the emergency department, the patient’s right knee was swollen; knee flexion was reduced to 30°. The right hip was painful on rotation. Serum laboratory values included a leukocyte count of 7,000/μL, hematocrit 33%, and erythrocyte sedimentation rate of 73mm/h. Plain radiograph images of the right hip did not show any abnormalities; the radiograph of the right knee showed suprapatellar effusion (Figure). Right knee aspiration yielded 115 mL of cloudy yellow fluid; laboratory tests showed a leukocyte count of 59,750/μL and protein level 5 g/dL; no crystals were noted. Results of routine bacterial culture of synovial fluid were negative. Amoxicillin was prescribed for a suspected septic joint and was taken for 1 week. Nine months later, the patient was reportedly asymptomatic and had returned to normal activity.

**Figure:**
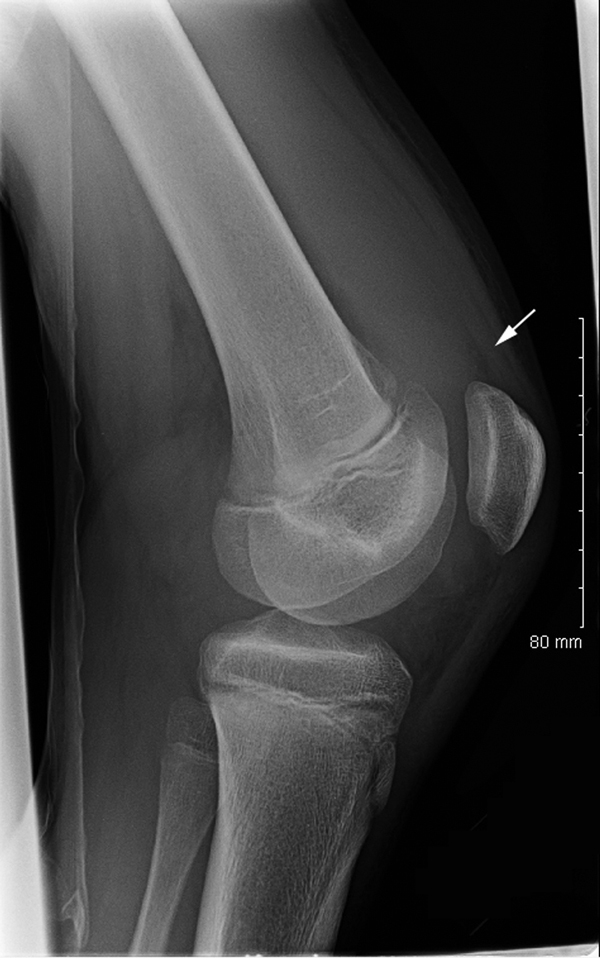
Lateral radiograph of right knee demonstrating suprapatellar effusion without acute osseous injury (arrow).

Right knee synovial fluid was sent to ARUP Laboratories (Salt Lake City, UT, USA); results were positive for the *B. burgdorferi* sensu lato *recA* gene by use of a proprietary qualitative PCR procedure. At the University of California, Irvine, we thawed another aliquot of synovial fluid, which had been frozen without cryoprotectant, and inoculated samples into BSK II medium (*8*). After incubation for 2 weeks at 34°C, no spirochetes were noted. We subjected another 100 μL aliquot to DNA extraction using DNeasy Blood and Tissue Kit and the QIAcube apparatus (QIAGEN, Valencia, CA, USA). We used multiplex quantitative PCR (qPCR) and primers and specific probes for the 16S ribosomal RNA genes of LB group species and for relapsing fever group species of *Borrelia* in 2 replicates as described by Barbour et al. (*9*). By qPCR, there were 18 gene copies of an LB group species in 1 replicate and 23 copies in the other. The qPCR results for relapsing fever group species, including *B. miyamotoi* and *B. hermsii*, which are enzootic in parts of California, were negative. We genotyped the *ospC* allele and 16S–23S intergenic spacer (IGS) using PCR amplification of each locus and direct sequencing as described by Travinsky et al. (*6*). Sequencing of the targeted PCR products showed that the *ospC* allele was type Hb and the IGS genotype was 13.

Two years of untreated relapsing pauciarticular arthritis of the knee and hip, a *B. burgdorferi*–positive Western blot, and laboratory detection of *B. burgdorferi* from synovial fluid by PCR in 2 different laboratories leads us to conclude that the patient had Lyme arthritis. This patient likely acquired the infection locally. The prevalence of *B*. *burgdorferi* in nymphal *I. pacificus* ticks (range 4%–10%) in Santa Cruz County, and >10% of the geographic area of the county is categorized as being at high acarologic risk for LB (*4*). To our knowledge, the combination of *ospC* allele Hb and IGS genotype 13 has been identified only in California to date (*6*,*8*). A type “H” *osp*C type was reported from synovial fluid from LB patients from the eastern United States (*10*), but in the absence of IGS determination, this was probably type Ha, which is more typical of that region (*8*). The addition of the IGS locus to *ospC* alleles provides a precise approach to characterize genetic diversity and potential origin of *B. burgdorferi* in human tissue.
